# Examining characteristics and sampling methods of phosphor dynamics in lowland catchments

**DOI:** 10.1007/s11356-024-33374-y

**Published:** 2024-04-29

**Authors:** Henrike T. Risch, Paul D. Wagner, Georg Hörmann, Nicola Fohrer

**Affiliations:** Department of Hydrology and Water Resources Management, Institute for Natural Resource Conservation, Kiel University, Kiel, Germany

**Keywords:** Lowland catchment, Phosphorus dynamics, Sampling strategies, Precipitation event sampling, Daily mixed sampling, Seasonal variation, Short-term concentration peaks

## Abstract

Despite over two decades since the EU Water Framework Directive have passed, achieving the desired water quality in German surface waters remains challenging, regardless of efforts to reduce phosphorus inputs and associated environmental impacts. This study aims at analyzing the characteristics governing the concentrations of four key water quality parameters (total phosphorus, orthophosphate, particulate phosphate, and suspended solids) in two lowland catchments: the 50 km^2^ catchment of the Kielstau, Germany, and its 7 km^2^ tributary, the Moorau, which are dominated by agricultural land use. To this end, different sampling methods, particularly high-resolution precipitation event-based sampling and daily mixed samples, are conducted and evaluated, and their effectiveness is compared. The identification of sources and characteristics that affect phosphorus and suspended sediment dynamics, both in general and specifically during heavy precipitation events, is one focus of the study. Over a 15-year period, increasing concentrations of these parameters were observed in daily mixed samples, exhibiting distinct seasonal patterns—higher in summer and lower in winter—consistent with lowland catchment behavior. Particularly during heavy precipitation events, the smaller catchment exhibits a more complex and less predictable response to chemical concentrations compared with the dilution effect observed in the larger catchment. The results underline the complexity of phosphorus dynamics in small catchments and emphasize the importance of event-based sampling for capturing short-term concentration peaks for all four parameters, particularly beneficial regarding measuring suspended solids. While daily mixed samples capture average phosphorus concentrations, event-based sampling is crucial for detecting short-term spikes, providing a more comprehensive understanding of phosphorus dynamics.

## Introduction

Although more than two decades have passed since the introduction of the EU Water Framework Directive, the desired “good status” could not be achieved in all German surface waters, despite various implemented measures to reduce agrochemical inputs and mitigate the associated environmental impacts. Studies indicate that the condition of the water network and major rivers is influenced by pollution sources in their immediate vicinity only to a limited extent and the impact of contaminants from smaller tributaries plays a significant role (Rutkowska et al. 2022; Steinhoff-Wrześniewska et al. 2022). This applies to lowland river systems that are characterized by ponds, lakes, and retention reservoirs.

The demand for agricultural products necessitates fertilization with phosphorus and nitrogen compounds to improve yields, as these are among the main limiting factors in plant growth (Tian et al. 2016). This additional nutrient amount on agricultural land also leads to negative impacts on the environment and particularly on water bodies (Addiscott et al. 1991; Ansari et al. 2011; Cui et al. 2020; Qi et al. 2023).

Nutrient pollution in water bodies can result from both, diffuse and point sources. The point sources are mainly connected to sewage. In rural areas, the limited cleaning power of the common small sewage treatment plants has a significant influence (Langergraber et al. 2018; Schranner 2014; UBA 2017). Diffuse sources from agriculture, especially inputs through erosion and drainage, make a significant contribution to phosphorus pollution in lowlands (Holsten et al. 2012; Trepel 2014). In lowland areas, the transport of water and nutrients is heavily influenced by its flat topography and shallow groundwater tables, as well as spatially heterogeneous land use (Krause et al. 2007; Lam et al. 2009; Lei et al. 2022; Schmalz et al. 2009).

Agricultural drainage, in otherwise relatively flat areas, greatly accelerates the movement of sediments and nutrients from these fields to receiving surface waters (Blann et al. 2009; Brendel et al. 2019; Skaggs et al. 1994) and plays a significant role in causing algal blooms and eutrophication problems in major surface water bodies worldwide (Bauwe et al. 2019; Rabalais and Turner 2019; Tiemeyer et al. 2009). Due to phosphorus being one of the most immobilized macronutrients in soil and its strong binding to soil particles (Arai and Livi 2013; Tian et al. 2016), heavy precipitation events have a major impact on the amount of phosphorus introduced into surface waters (Waller et al. 2021). Such extreme precipitation events are occurring with increased frequency due to climate change (Myhre et al. 2019) and can be observed primarily in the summer months often associated with convective air movements (DWD 2022; LAWA 2018; Myhre et al. 2019).

Considering the spatial and temporal fluctuations in water quality, ensuring sample representativeness becomes a crucial concern while devising an appropriate monitoring strategy and frequency to assess the long-term water quality trends and the efficacy of mitigation measures. Increasing the precision and accuracy of measurements can be achieved by selecting a suitable sampling time interval tailored to specific parameters (Skeffington et al. 2015). Historically, monthly or fortnightly grab sampling has been commonly employed due to its cost-effectiveness (Ferreira et al. 2007). However, this approach may overlook short-term, high-concentration peaks that arise during infrequent high-flow events throughout the year, especially with regard to suspended solids (SS) and total phosphorus (TP) and a more frequent sampling is necessary (Bowes et al. 2003; Brauer et al. 2009; Cassidy and Jordan 2011; Horowitz 2008; Sun et al. 2022; Waller et al. 2021).

To capture temporal changes in TP concentrations, especially in smaller agricultural catchments with fast response to rainfall, continuous monitoring at high temporal resolution (e.g., hourly and sub-hourly sampling) is essential (Cassidy and Jordan 2011). This is crucial because intense short-duration rainfall events can significantly contribute to the total diffuse transfer of TP from soil to water (Cassidy and Jordan 2011).

Another strategy to enhance load estimates is flow proportional sampling, which has been investigated and integrated in agricultural monitoring catchments in Northern Europe. This method ensures that sampling efforts are proportional to the flow rate, enabling a more detailed estimation of pollutant loads during varying flow conditions (Kyllmar et al. 2014).

To comprehensively understand and monitor the phosphorus dynamics within a lowland catchment, the following research questions are addressed:

1. What are the sources and characteristics influencing phosphorus dynamics within a lowland catchment?
2. How does heavy precipitation influence phosphorus and suspended solids discharge in two different sized lowland catchments?
3. Could continuous daily sampling benefit from additional precipitation event-based sampling with a high temporal resolution?

## Materials and methods

### Study area

**Fig. 1:**
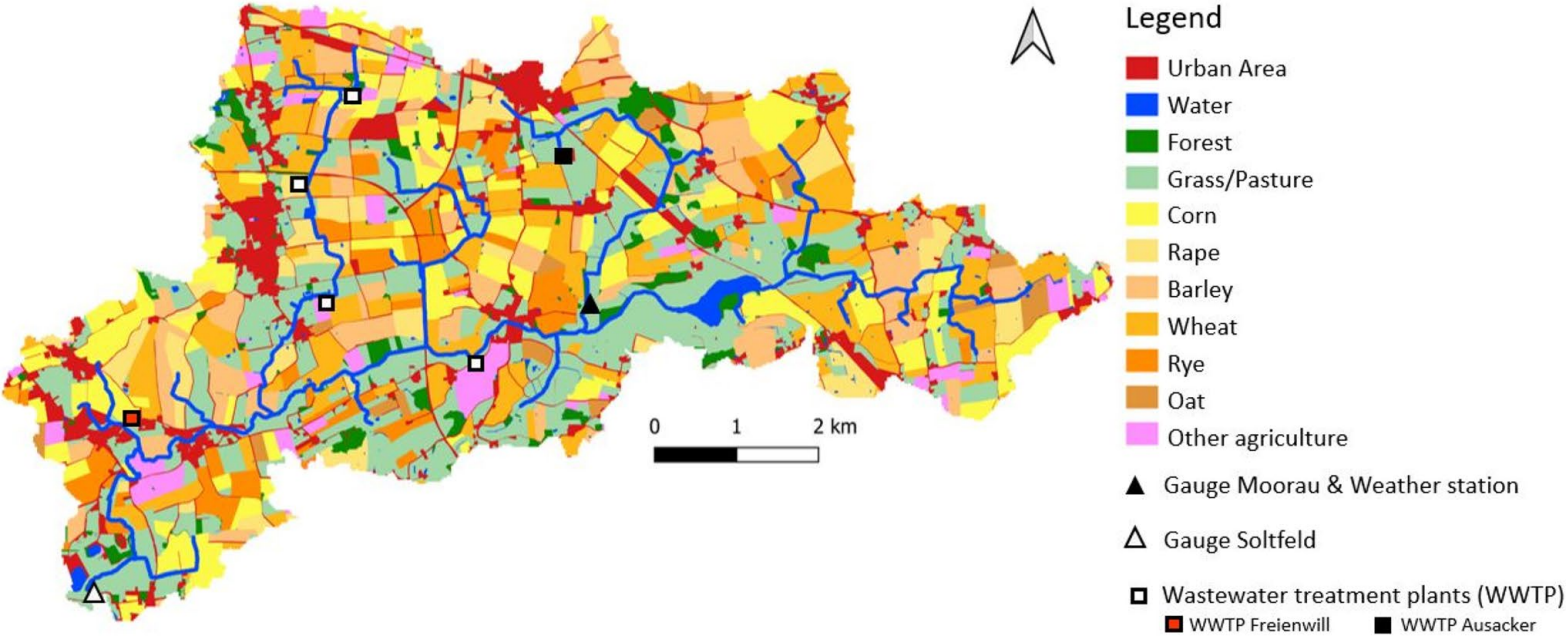
Land use map from 2021 of the Kielstau catchment with the sampling locations, the weather station and the wastewater treatment plants

The Kielstau catchment is a rural lowland catchment in northern Germany with a catchment area of about 50 km^2^ (Fohrer et al. 2014; Wagner et al. 2018) (Fig. 1). The Kielstau river drains towards the west and into the Treene river. It is a gravel lowland stream and has a total length of about 17 km, flowing through rural areas with a small number of minor communities, detached farms, and no major industry. It originates north of Sörup at an altitude of 45 m above sea level. Water quality measurements are taken in the Kielstau catchment since 2005 by the Department of Hydrology and Water Management at Kiel University (Femeena et al. 2019; Sun et al. 2022; Wagner et al. 2018). Moreover, the area is recognized as a UNESCO ecohydrology reference project since October 2010 (Fohrer and Schmalz 2012; UNESCO 2011).

The catchment area is situated in the young moraine landscape of Schleswig–Holstein Morainic Uplands and the topography is comparatively even with heights between 27 and 78 m above sea level (LVERMA 2006). The dominant soil types in the area are primarily Luvisols with a distribution of Gleysols and Cambisols in central area of the catchment (BGR 1999). The mean annual precipitation of 918.9 mm and a mean annual temperature of 8.2 °C characterize a temperate climate (Flensburg station, period 1961 to 1990, DWD 2021). At the Soltfeld gauge, the outlet of the catchment area, an average water level of 50 cm and an average discharge of 0.46 m^3^/s were measured between the years 2007 and 2021 (MELUR 2023).

A land use map based on a field survey in summer 2021 shows that agricultural use with arable farming (63.7%) and grassland (20.3%) dominates in the catchment area (Fig. 1). Crop rotations are generally used, resulting in frequent changes within the areas of arable farming. Forest (3.3%), orchards and horticulture (0.5%), settlement and traffic areas (10.5%), and water areas (1.6) account for smaller areas. To increase agricultural production, subsurface tile drainages are used and estimated to cover 38% of the entire catchment (Fohrer et al. 2007).

The Kielstau river hast two major tributaries, the Moorau and Hennebach rivers (MELUR 2023; Schmalz and Fohrer 2010). About 5 km downstream from the spring it flows through Lake Winderatt, surrounded by protected areas of the “Winderatter See” foundation that are mainly used for moderate grazing (Fohrer and Schmalz 2012; Förderverein Winderatter See – Kielstau e.V. 2017).

Six wastewater treatment plants drain into the Kielstau and its tributaries, and two biogas plants are located within the catchment (Lei et al. 2019). The treatment plants Ausacker and Freienwill are located on the main stream. Wastewater treatment plant Husby is situated at the beginning of the Moorau tributary, while Hürup Nord, Hürup Weseby, and Hürup Süd are located along the Hennebach tributary (Fig. 1).

### Precipitation and runoff measurements

Water level and discharge are recorded by the state agency of Schleswig–Holstein at the Soltfeld gauge since 1985 and are available in daily resolution (MELUR 2023).

There is a weather station maintained by the Department of Hydrology and Water Resources Management at the Gauge Moorau sampling site since 2010, which records precipitation with a 10-min resolution. The distance from the weather station to the Gauge Soltfeld is 5 km. In line with the German Weather Service classifications for rain intensity, the recorded rainfall can be categorized as follows: light precipitation (in 60 min < 2.5 mm, in 10 min < 0.5 mm), moderate precipitation (in 60 min ≥ 2.5 mm and < 10.0 mm, in 10 min ≥ 0.5 mm and < 1.7 mm), heavy precipitation (in 60 min ≥ 10.0 mm, in 10 min ≥ 1.7 mm), and extreme precipitation (in 60 min ≥ 50.0 mm, in 10 min ≥ 8.3 mm) (DWD 2024).

### Sampling

#### Sampling points

Two points in the Kielstau catchment were selected for sampling. The first sampling point is situated at the outlet of the Moorau tributary upstream of its confluence with the Kielstau River and is referred to as Gauge Moorau in the following. The Moorau tributary flows through a 2-km-long underground pipe system before it emerges south of Husby, about 12 km southeast of Flensburg. After surfacing, it is an approximately 5-km-long stream flows into the Kielstau east of the settlement Ausacker. At the Gauge Moorau sampling location, samples are collected at a bridge, where the river traverses a concrete pipe with a diameter of 100 cm, subsequently transitioning into an open channel. The second sampling point is located at the outlet of the entire Kielstau catchment and is named Gauge Soltfeld. The width of the river at the sampling location measures 310 cm with little variation at the recorded water levels. It is located in Großsoltbrück, which is a district of the municipality of Großsolt in the district of Schleswig-Flensburg in Schleswig–Holstein. It is surrounded by extensively used farmland that is sporadically also used for grazing cattle.

#### Sampling strategy

##### Daily mixed samples

Starting from 2006, a refrigerated auto-sampler (Refrigerated Maxx-Sampler SP 5 S) is installed at the second sampling point (Gauge Soltfeld) at the outlet of the Kielstau catchment. This sampler collects automated mixed samples (100 ml per 70 min over a 24-h period starting at 12:00 a.m.) directly from the river on a daily basis. The samples are stored at a temperature of 4 °C and later transported to and analyzed in the laboratory of the Department of Hydrology and Water Resources Management at Kiel University.

##### Event-based samples

The event-campaign ran during the hydrological summer half-year 2021 from May to November. For this campaign, two different samplers were used at the two sites. At the Gauge Soltfeld, the sampling is based on technology from Teledyne ISCO ©, with the ISCO sampler, a signature flow meter and a TIENet Model 350 Area Velocity Sensor. The measurement setup at the Gauge Moorau consists of a combination of a sampler and floating trigger from MAXX GmbH © and a velocity sensor as well as the Avelour 6 software from IJINUS ©. In addition, an SMS modem was connected to the trigger mechanism at Gauge Moorau and informed in real time of the sampling progress so that the pick-up and analysis of the samples in the laboratory were carried out promptly. The flow velocity gauges at both measuring locations took measurements of the water level at 5-min intervals. This makes it possible to automatically trigger a logarithmically structured sampling with high temporal resolution at both locations if the water level rises by at least 2 cm in less than 2 h.

**Fig. 2:**
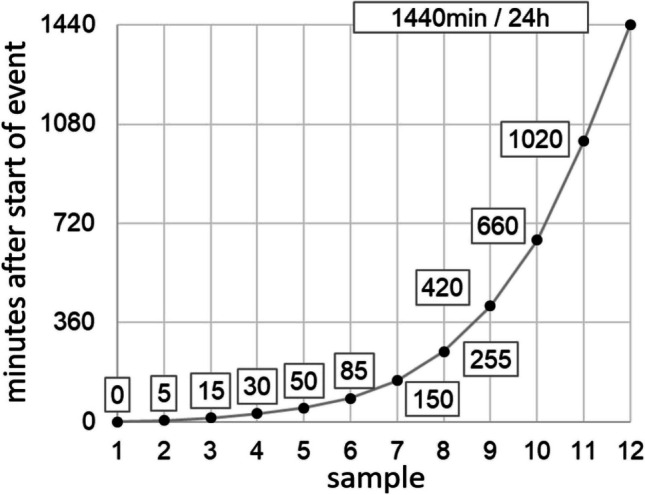
Distribution of sampling time in minutes after start of sampling event

Twelve water samples of about 1800 ml each were taken over the course of 24 h per event using this logarithmic sampling method (Fig. 2). The data set per event includes the water level data 24 h before the start of an event, the 24 h of sampling and 1 h after the end of the event. In total, 49-h periods are analyzed for each event.

#### Water quality analysis

In the laboratory of the Department of Hydrology and Water Resources Management at Kiel University, the daily mixed and event-based samples are assessed using the German standard procedure for water analysis (DEV). Orthophosphate (OP) (DEV D11 and DIN 1189) and total phosphorus (TP) (DEV H36, DEV D11, and DIN 1189) are measured with the ammonium molybdate spectrophotometric method at 880 nm. Particulate phosphate (PP) was calculated as the difference of TP and OP. In addition, the suspended solids (SS) are determined by 45 µm filtration of 1 l sample volume. This relatively small sample volume affects the measured values, especially in SS concentrations. This introduces some uncertainty with regard to the absolute values and their interpretation. However, the setup remains consistent for the daily and the event-based methodology so that the samples are comparable among themselves.

### Water quality classification

The Working Group on Water Issues of the Federal States and the Federal Government (LAWA) has developed background and orientation values for water quality parameters in relation to the surface water type groups (Table 1) in part B of the Conceptual Framework (RaKon) for the establishment of monitoring programs and the assessment of the state of surface waters. When the orientation value is violated, the phosphorus concentration assumes an order of magnitude that generally no longer permits a good ecological status of the body of water, even if the orientation value of no other parameter is violated (LAWA-RaKon 2015). The background value is the threshold for good ecological status. The background and orientation value for the Kielstau, a gravel lowland stream, are provided in Table 1 (LAWA-RaKon 2015).

**Table 1:** Reference values of the LAWA-RaKon according to river type 17 (LAWA-RaKon 2015)

	Total phosphorus	Orthophosphate
Background value	0.05 mg/l	0.02 mg/l
Orientation value	0.10 mg/l	0.07 mg/l

### Statistical methods

A comprehensive trend analysis was conducted for the data gathered at the main outlet of daily mixed samples (2007–2021). For a division of the time period in summer and winter season, the months April to September are classified as the summer half-year, and the months from October to March are the winter half-year. To characterize the water and matter balance at both locations, statistical parameters, frequency distributions, correlations, as well as cross-correlations of the parameters are examined. The Pearson’s correlation coefficient is used. The water level is used as the second parameter for the cross-correlation. Linear regression is used to analyze the changes in the water and matter balance at the sampling points. The slope of the regression line reflects the trend in the time series. The significance of the trend is determined at the 5% significance level. In addition to the individual trend analysis for each event, a trend analysis is performed for the entire data set collected at each sampling site. All analyzes were performed with the statistical software R 4.2.3 (R Core Team 2023).

## Results and discussion

### Daily mixed samples

**Fig. 3:**
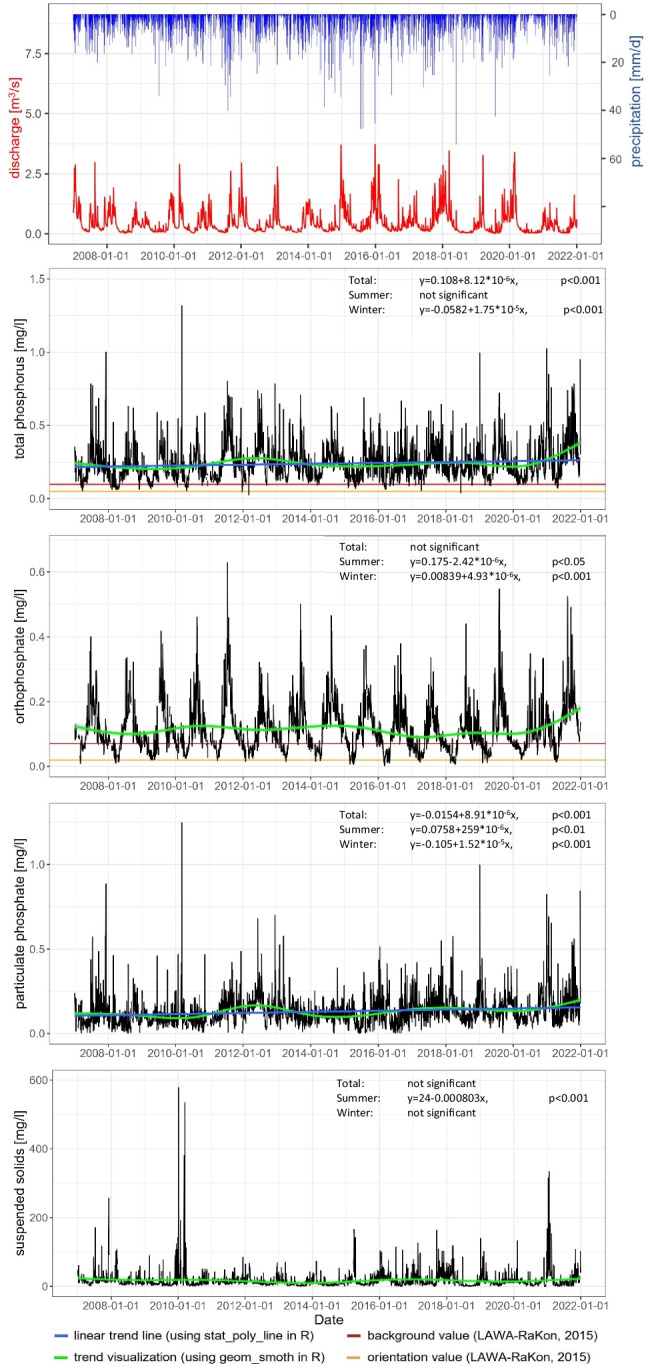
Dynamics of discharge and the daily mixed sample data (total phosphorus, orthophosphate, particulate phosphate and suspended solids 2007–2021) with significant linear trend lines, regression equation and p value for the entire period, summer and winter half-year, trend visualization and LAWA-RaKon (2015) limit values (Table 1)

The analysis of the long-term data series in the Kielstau catchment at Gauge Soltfeld from 2007 until 2021 showed an increase in phosphorus concentrations. Over the 15-year period, the linear regressions for PP and TP are statistically significant (*p* < 0.001). However, no significant increase of SS could be determined in the Kielstau at Gauge Soltfeld (Fig. 3). The seasonal linear regression lines show an increase in all phosphorous parameters in winter, whereas the summer shows a significant concentration increase only in PP (Fig. 3).

The average TP and OP concentrations are higher in summer than in winter. This seasonal pattern, with higher concentrations in summer than in winter, in the Kielstau was also found by Wagner et al. (2018). Other researchers also documented similar seasonal effects in comparable lowland catchments in north Germany (Bauwe et al. 2019; Bitschofsky and Nausch 2019) and elsewhere (Ballantine et al. 2008, May et al. 2001, Schmalz et al. 2016 2015, Yates and Johnes 2013, Zieliński and Jekatierynczuk-Rudczyk 2015). These effects are frequently linked at least partially to point sources, which experience more dilution during periods of high flow than during periods of low flow (Abbott et al. 2018; Bowes et al. 2003; Yates and Johnes 2013). Diffuse inputs from mineral P fertilization in connection with heavy rainfall can also contribute to these seasonal changes (Wagner et al. 2018).

A stronger increase in the concentration of TP can be found from 2020 onwards. Also, in the case of OP, the concentration increased, especially in 2020 and 2021 (Fig. 9 in the Appendix). Among all the calculated regressions of the daily mixed samples, the OP concentrations during the winter period from 2017 to 2021 (Fig. 9) exhibit the highest coefficient of determination (*R*^2^ = 0.26). This indicates that the linear regression model can explain 26% of the variability in OP concentrations. However, it is important to note that while this *R*^2^ value is the highest among the measured datasets, it remains relatively low. The calculated PP also showed an increase in concentration trends over the entire measurement period (Fig. 3). One of the impact factors for this increasing trend could be the increase in population in the district Schleswig-Flensburg, in which the Kielstau catchment is located. The population in 2007 was 199.101, which increased to 203.779 by the year 2021 (Keller 2023).

As explained in Lei et al. (2019), six wastewater treatment plants drain into the Kielstau river. One of these plants is the Freienwill wastewater treatment plant which operates under 2700 EWG (population equivalent). The Freienwill wastewater treatment plant is currently being expanded to approximately 6000 EGW until January 2024, due to a merger with the municipality of Hürup with additional wastewater and due to population increase in the Freienwill region (D. Behnemann, personal communication, June 06, 2023), supporting the population increase noted by Keller (2023).

**Fig. 4:**
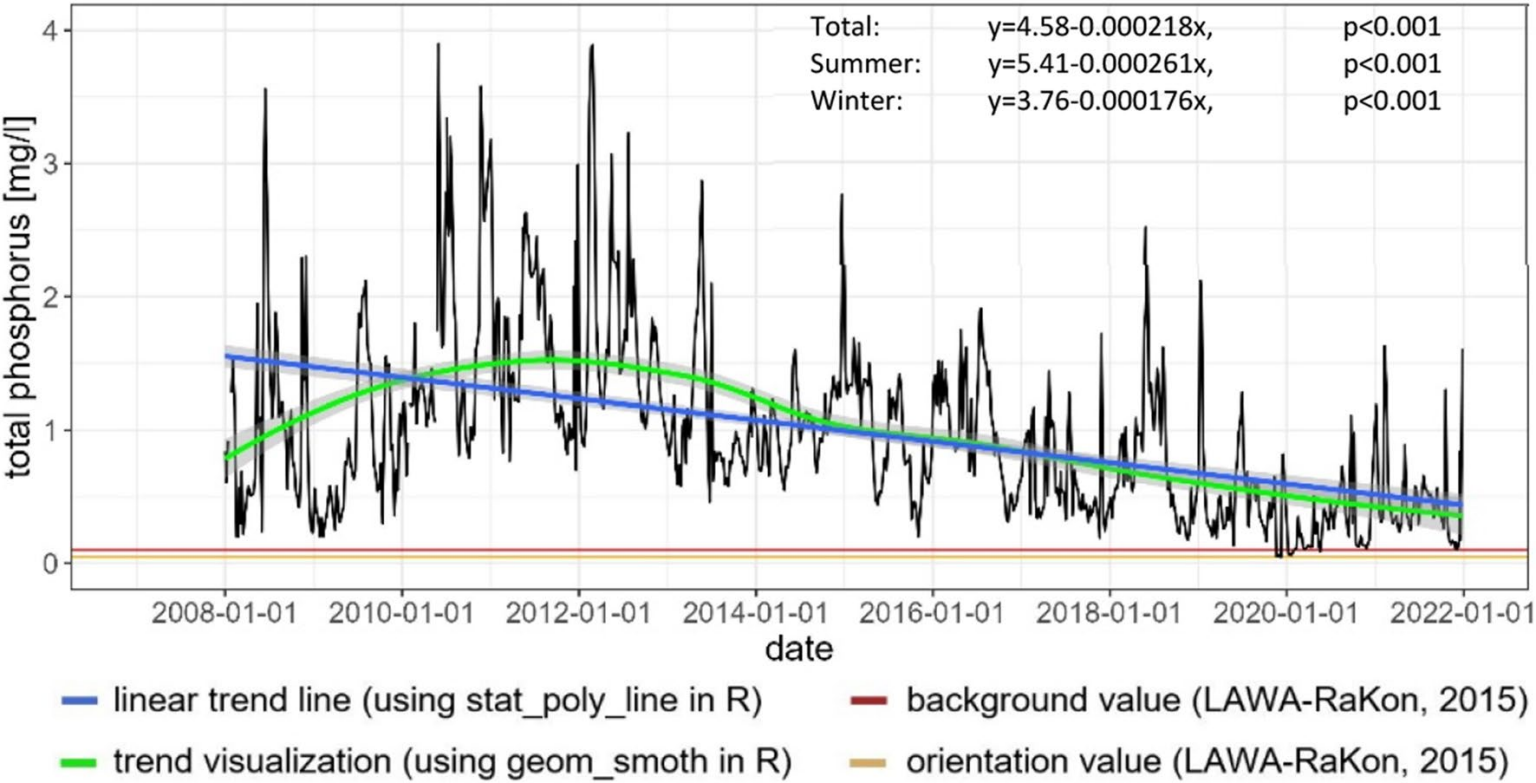
Dynamics of total phosphorus concentrations of the Freienwill wastewater treatment plant outlet (2008–2021) (Nord 2023b) with significant linear trend lines, regression equation and p value for the entire period, summer and winter half-year, trend visualization and LAWA-RaKon (2015) limit values (Table 1)

Between the years 2008 and 2021, the measured annual wastewater volume at Freienwill increased from annually 85,987 m^3^ to 114,968 m^3^ (Nord 2023b) and concentrations from 4 mg/l to 0.05 mg/l were released into the Kielstau (Nord 2023b). A calculated significant linear regression (*p* < 0.001) shows a decrease in released TP concentration over the total summer and winter periods. Even with a concentration decrease, the total of TP drained into the river could have increased and could have contributed to the increase in phosphorus concentrations over the years (Fig. 4), but it is unlikely that it is the dominant reason for the increase of phosphorus concentration at Gauge Soltfeld.

Furthermore, the land use of 2021 (Fig. 1) is a factor affecting water quality, because agricultural use with arable farming (63.7%) and grassland (20.3%) dominates in the catchment area. The dissolved form of inorganic phosphorus in the soil, which is readily available for plant uptake, constitutes only a small fraction (Kruse et al. 2015). As a result, agricultural production systems usually rely on fertilization to supplement the accessible phosphorus for optimal plant growth.

In the light of the two biogas plants situated in the catchment, the utilization of biogas digestates as fertilizers is probable. Compared with untreated manures, biogas digestates generally have lower dry matter content, influenced by co-substrates used (Kluge et al. 2008; Pötsch et al. 2004). The TP content in digestates varies (Lfl 2012). But it usually ranges from 0.04% to 0.08% of fresh mass, similar to untreated manures (Kluge et al. 2008). Phosphates in all types of digestates have long-term effectiveness similar to mineral fertilizers (Bachmann 2013; Lfl 2012). This and the fact that the biogas plants are not a recent addition (older than 2016) indicate that they are also not the main contributor of increased phosphorus concentrations.

A significant phosphate loss is generally caused by soil erosion, i.e., horizontal displacement of soil particles by water or wind (Christoffels 2013; Galler 2008; Holsten et al. 2012; Trepel 2014). Climate change can alter plant growth stages, including crops, impacting ground cover and increasing erosion risks. Drought-induced vegetation gaps and dry soil surfaces worsen erosion, especially in sandy-soil coastal areas, where wind is an additional factor. Escalating spring and summer droughts heighten wind erosion risks (UBA 2019). In lowland catchments with little slope, which generally has comparably a low risk of erosion but high levels of agricultural use and drainage density, seepage-related paths via the intermediate runoff and the groundwater can also dominate (Kiesel et al. 2009, VDLUFA 2001). The phosphorus discharge via seepage water increases with the following factors: increasing phosphorus saturation of the topsoil, increasing amount of seepage water, increasing proportion of coarse pores, and decreasing seepage distance (Fischer et al. 2017). The amount of seepage water in turn depends on the amount of precipitation and the field capacity. The seepage distance can be shortened by drainage laid close to the surface (Skaggs et al. 1994; VDLUFA 2001). In 2021, maize, rapeseed, wheat, barley, and rye were primarily cultivated in the catchment. Grain crop rotations are predominant. The most is the alternation of wheat and maize, which occurs on approximately 13% of the area in 2020 and 2021. Maize is also cultivated extensively with barley or rye. If good professional practice and maintenance fertilization (Wiesler et al. 2018) is applied, a shift of phosphate to the lower soil layers is low (Galler 2008).

From 2017 to 2021, the concentration of only one TP sample was below the background value, and the concentration of only 21 samples were under the LAWA-RaKon (2015) orientation value. In 47 cases, OP concentrations were below the background and 647 samples were below the orientation value (Table 1 & Fig. 9). The ecological status of the Kielstau can only be classified as moderate in this period. The classification of the measured data of 2021 specifically showed an increasing number of violations of the orientation and background value (Figs. 3 and 9). Because the classification of the ecological status in the case of non-compliance with an orientation value is an indication of a specific, ecologically effective insufficiency, the Kielstau has to be classified as moderate condition, missing the desired good ecological status (LAWA-RaKon 2015).

### Event-based samples

**Fig. 5:**
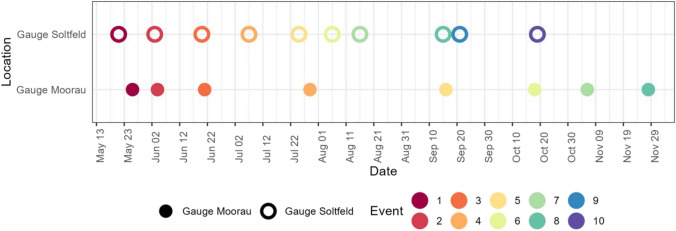
Distribution of events at both gauges over the 7 months sampling period in 2021

**Fig. 6:**
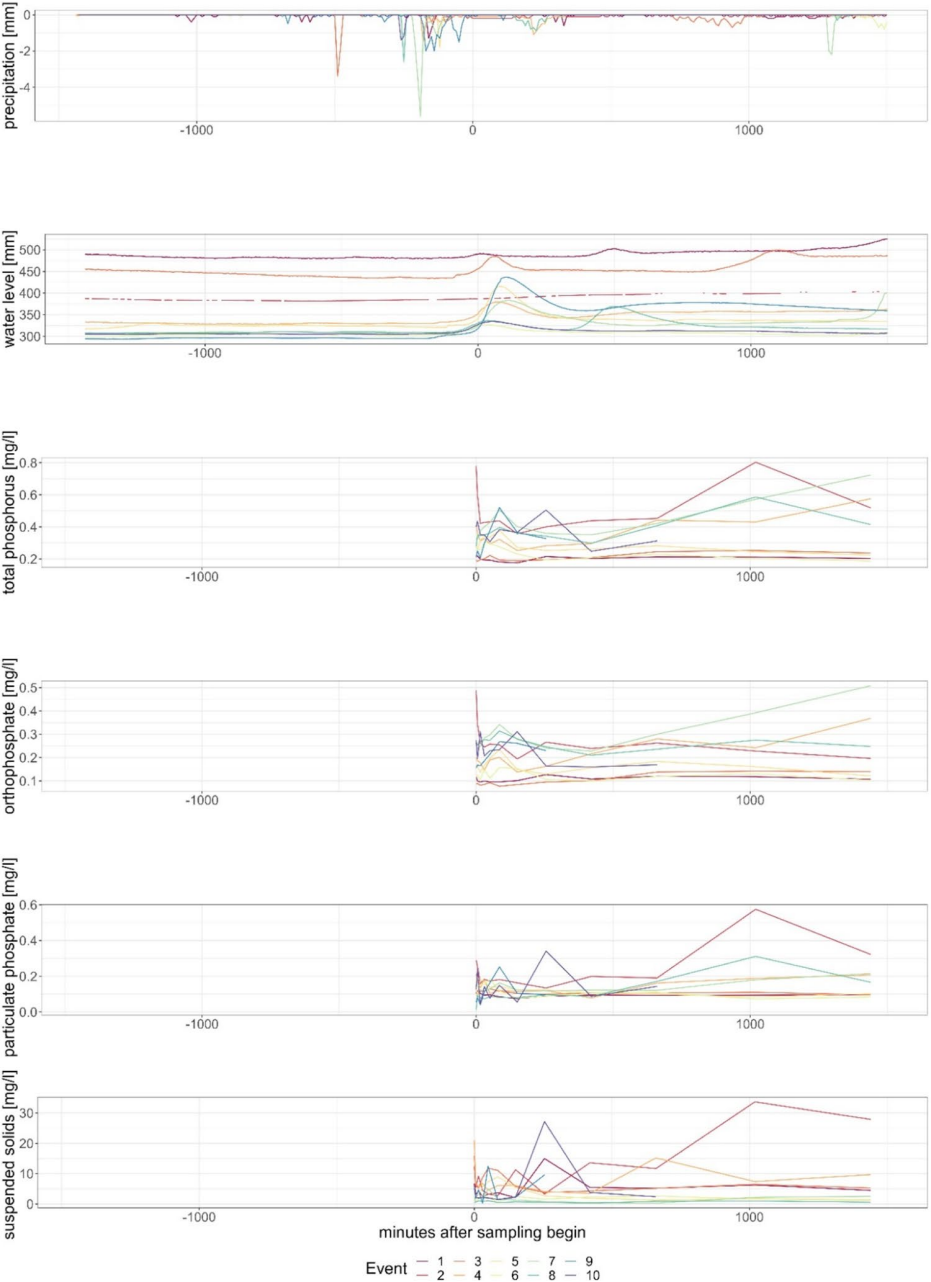
Events at Gauge Soltfeld with precipitation, water level, total phosphorus, orthophosphate, particulate phosphate and suspended solids in relation to the event-based sampling start

**Fig. 7:**
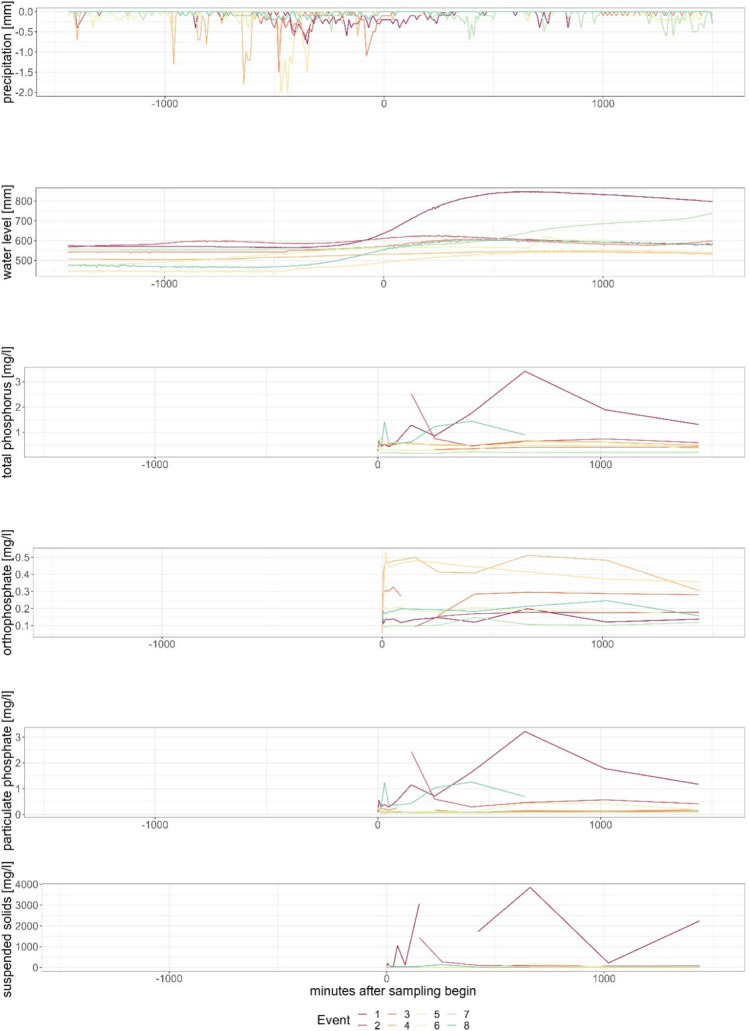
Events at Gauge Moorau with precipitation, water level, total phosphorus, orthophosphate, particulate phosphate and suspended solids in relation to the event-based sampling start

During the seven-month sampling period from May to November at both stations, event-based samples were collected. At Gauge Moorau, data was collected for eight events and at Gauge Soltfeld for ten events. The events at Gauge Soltfeld range from 21 May until 19 October. The events at Gauge Moorau occurred between 26 May and 28 November with fewer events in the summer months (Fig. 5). The time-wise closest events were event 8 at Gauge Soltfeld and within 5 h Event 5 at Gauge Moorau. Events 2 at Gauge Soltfeld and Gauge Moorau were triggered close together (22 h difference). On no occasion was the sampler triggered at the same time at both gauges; therefore, the results for both gauges are presented separately (Figs. 6 and 7).

One impact factor for the difference in reaction at both gauges is the size of the different corresponding catchments. The catchment response time greatly varies for different sized catchments with travel time of the water to the outlet gauge, with usually faster responses and higher impact in small catchments (Black et al. 2021; Gericke and Smithers 2014). However, the water level increased earlier at Gauge Soltfeld, which has the larger catchment area, as compared with Gauge Moorau. This shorter trigger can be partially attributed to the fact that a 2-cm increase in water level is achieved faster at Gauge Soltfeld due to the larger catchment area. Another contributing reason is that the catchment typically receives precipitation from the prevailing west winds so that the water level also rises earlier in the west, where Gauge Soltfeld is located. Unfortunately, the effect of rainfall distribution cannot be proven with our data, as there is only one precipitation gauge in the entire Kielstau catchment. The weather station is located central near the outlet of the Moorau catchment at Gauge Moorau. A rain event could have already started in parts of the catchment contributing to discharge before being recorded at the measurement station Moorau (Zhang et al. 2021a, 2021b).

At the start of event 2 in Gauge Soltfeld, east winds were recorded at the weather station Moorau, changing to southeast winds until the start of Event 2 at Gauge Moorau. This would support the earlier statement of wind direction and the corresponding possible precipitation patterns over the catchments influencing the response time until a water level change occurs, starting the sampling process. Rainfall in the eastern part of the catchment contributes to streamflow of the Kielstau, whereas streamflow at the Moorau is generated in the northern part of the catchment. During the start of Event 8 at Gauge Soltfeld, south winds were dominant, changing direction to dominant southwest winds, indicating that big precipitation patterns should hit Kielstau and Moorau patterns in a similar timeframe due to their latitude. Generally, the wind data from this station shows a large variability in wind direction and speed over the seven-month measurement period.

The strongest rise in water level at Gauge Soltfeld was observed during event 8, occurring after approximately 2 h of precipitation. The triggering precipitation events at Gauge Soltfeld were consistently observed to be -180 min bevor trigger of the sampler (within 3 h). The only exception found at event 2 had a delay of approximately 5.5 h, corresponding the rain event visible at approximately -330 min before the trigger. The difference in reaction time can be attributed to the speed and direction of the cloud movement, to rainfall intensities, as well as to different initial hydrologic conditions in the catchment, e.g., in soil moisture. However, the water levels at Gauge Soltfeld begins to rise approximately 2 h before the sampler was triggered, suggesting a consistent pattern in the timing of precipitation and water level changes. Events 1, 2, and 3 at Gauge Soltfeld started with significantly higher water levels 24 h before the trigger of the sampler (Fig. 6). This corresponds with what is considered part of the high flow season before the beginning of summer (Abbott et al. 2018; Bitschofsky and Nausch 2019).

At Gauge Moorau, many discontinuous precipitation events were observed. The strongest rise in water level occurred during event 1, where the water level was increased by 270 mm. This is associated with a rainfall sum of 5.3 mm starting 530 min before the event and continuing until 330 min before the event. From the middle of this strong rainfall period until a noticeable water level change and a trigger of the sampler, 425 min, i.e., approximately 7 h, passed. During the entire scattered rainfall event from minute 530 before and 320 after the start of the event, a total amount of 18 mm rain was recorded, contributing to the extreme rise in water level. After scattered rain events, Gauge Moorau exhibited a slower rise in water levels compared with short and heavy precipitation events. The behavior of chemical concentrations varied significantly between different events at Gauge Moorau (Fig. 7). This indicates a multitude of contributing factors. One of these factors, albeit less influential than at the Gauge Soltfeld, the precipitation movement over the Moorau catchment could have some impact. A further factor that was investigated due to its impact on the lag time is the soil moisture content (Haga et al. 2005). Using the German drought monitor (GDM) (Boeing et al. 2022) as a base, the volumetric data soil water content in the Kielstau area was modeled and used as an indicator of soil moisture before the rain events. However, the soil moisture data could not explain the different dynamics of water level and phosphorus concentrations.

It has to be noted that none of the precipitation events that occurred during the sampling period met the criteria for extreme rainfall (minimum of 50 mm of precipitation per hour or 8.3 mm per 10 min) (DWD 2024). The measured rainfall events during the sampling period had a maximum of 9.2 mm per hour, coming short of the 10 mm per hour cutoff (DWD 2024) for heavy precipitation. However, several short-term peaks between 1.7 mm and 8.3 mm per 10 min were recorded and can still be classified as heavy precipitation events at Gauge Soltfeld and Gauge Moorau.

Correspondence between the concentration increase of SS and the phosphorus fractions is often visible, but especially dominant in event 2 at Gauge Soltfeld and event 1 at Gauge Moorau (Figs. 6 and 7). In terms of phosphorus fractions and SS concentrations, Gauge Soltfeld showed significantly lower levels compared with Gauge Moorau, indicating a dilution effect of phosphorus concentrations and potentially improved water quality. The Gauge Soltfeld exhibited significantly faster and shorter water level changes compared to Gauge Moorau. The triggering rain events at Gauge Soltfeld were consistently within 3 h before the start of the sampler, while Gauge Moorau mostly reacted to discontinuous precipitation events (Figs. 6 and 7).

The wastewater treatment plant Ausacker drains into the Moorau close to its source. In the year 2021, a total amount of 11161 m^3^ wastewater was cleaned in the facility and three analyses of TP at the outlet into the river were conducted. On 8 April, a concentration of 2.07 mg/l, on 20 July 5.32 mg/l, and on 21 October 6.64 mg/l of TP were measured (Nord 2023a). A comparison to the larger Freewill treatment plant in the catchment shows that Ausacker has significantly higher concentrations at the outlet. Freienwill’s concentrations of TP are continuously between 0.1 and 1.7 mg/l (Nord 2023a, 2023b). This indicates that the treatment plant Ausacker with its higher TP concentrations is contributing to the high phosphorus concentrations found at Gauge Moorau.

With regard to the sampling setup, the trigger adequacy of a two-centimeter water level change within 2 h as a suitable approximation for reflecting heavy rain events seems fitting, because the sampling process was triggered on the rising part of the water level curve and the samples were taken during the high water level peaks. However, due to the significant time delay between precipitation events and sampling start, any higher concentration peak that may have occurred without a corresponding water level change was not captured. Generally, the behavior of chemical concentrations varied greater between different events at Gauge Moorau than at Gauge Soltfeld. This suggests a more complex and less predictable system in the smaller upstream catchment compared to the larger catchment, in which different effects may have balanced each other and concentrations were diluted. However, not just both sampling points, but also each individual event greatly differs from the others in terms of behavior and characteristics so that the prediction of future events is difficult.

Distinct differences in correlations were observed between Gauge Moorau and Gauge Soltfeld. Gauge Moorau exhibited a higher number of significant correlations, suggesting a greater level of interdependence between variables compared to Gauge Soltfeld. At Gauge Moorau, five correlations have an R-value above 0.5, whereas Gauge Soltfeld only achieved correlations above 0.5 in two instances. The largest positive correlation at Gauge Soltfeld was found between PP and TP, with a correlation coefficient of 0.84. At Gauge Moorau, a stronger correlation coefficient of 0.96 was observed between TP and PP (Fig. 10 in the Appendix). Suspended sediments are strongly positively correlated with TP at Gauge Moorau (*r* = 0.798), whereas the correlation is lower at Gauge Soltfeld (*r* = 0.296). Therefore, it can be assumed that the entry path of phosphorus in the Moorau catchment is primarily erosion of sediments. In the larger Kielstau catchment, this effect is not as obvious, possibly due to dilution effects. The very low correlations between precipitation and water level can be attributed to lag time and catchment response time at both sampling points. The cross-correlation value is relatively low (0.32), with peak correlation achieved after a lag of 2 days (Wagner et al. 2018). The relatively low correlations between the water level and chemical concentrations, especially at Gauge Soltfeld, are affected by the short water level peaks. Concentration peaks during the events are most common at a rising, but still low water level and after the water level peak. The longer more consistent water level rise at the Gauge Moorau possibly led to higher correlation between water level and the chemical concentrations. The generally lower correlations at the Gauge Soltfeld and larger mixed signal can also at least partially be attributed to the bigger catchment area and the increased distance from the weather station.

### Comparison of daily mixed and event-based samples in 2021

**Fig. 8:**
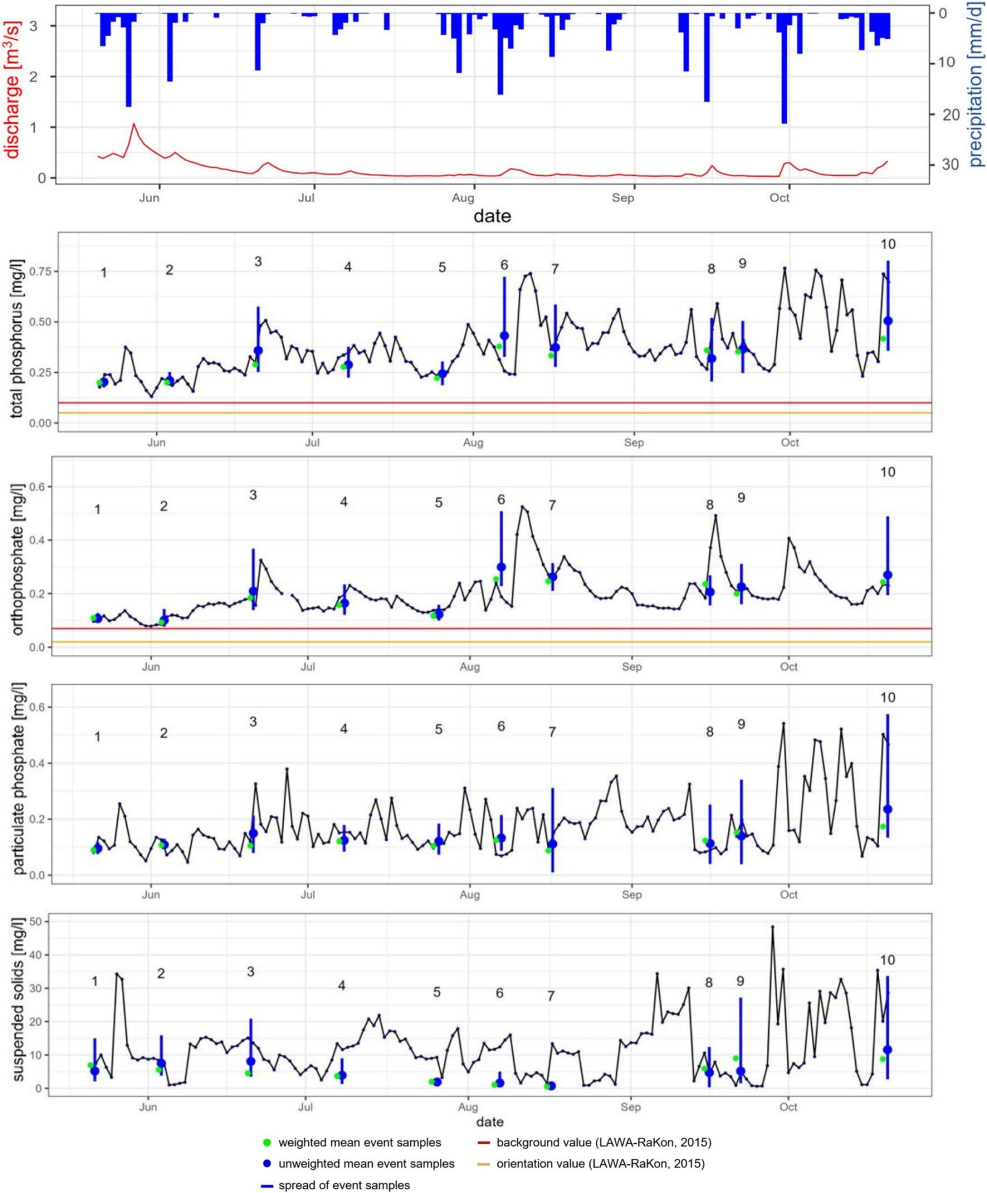
Dynamics of precipitation, discharge and the daily mixed sample data (total phosphorus, orthophosphate, particulate phosphate and suspended solids) over the measurement period with the weighted and unweighted mean values of the event samples (total phosphorus, orthophosphate, particulate phosphate and suspended solids) and the LaWa-RaKon (2015) limit values (Table 1) at Gauge Soltfeld

**Fig. 9:**
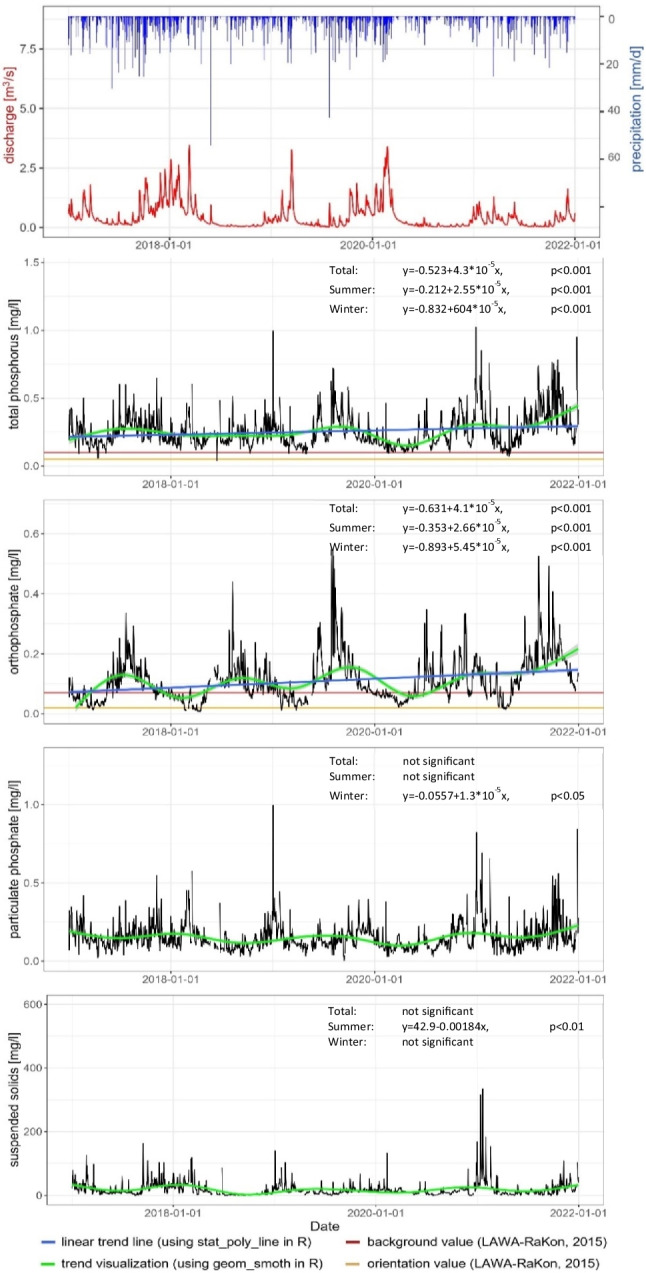
Dynamics of discharge and the daily mixed sample data (total phosphorus, orthophosphate, particulate phosphate and suspended solids 2017–2021) with significant linear trend lines, regression equation and p value for the entire period, summer and winter half-year, trend visualization and LAWA-RaKon (2015) limit values (Table 1)

**Fig. 10:**
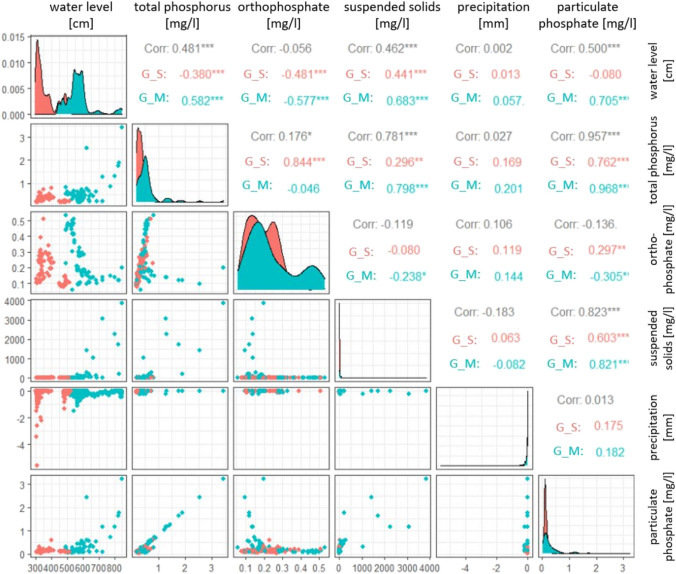
Scatter plot matrix with the density distribution of the precipitation, water level, total phosphorus, orthophosphate, particulate phosphate and suspended solids data in the diagonal, scatter plots in the lower left and the correlations (r) in the top right. The Gauge Soltfeld (G_S) data is represented in red, while the Gauge Moorau (G_M) data is depicted in blue. Grey represents correlations for the entire data set. The precipitation data is inverted

The event-based samples are compared to the daily mixed samples at Gauge Soltfeld. To this end, weighted (green) and unweighted (blue) mean values of the event samples are calculated and compared to the dynamics of the daily mixed samples at Gauge Soltfeld (Fig. 8). The first data point from event 1 is starting on 21 May, with the beginning of the sampling at 16:40. The 12 samples in the following 24 h are taken following a logarithmically structured sampling scheme so that the time difference between the samples is steadily increasing (Fig. 2). A mean value calculation and a weighted mean value with the different times incorporated in the calculation were carried out.

At Gauge Soltfeld, the concentrations remained consistently above both the background and the orientation value of the LAWA-RaKon (2015), indicating persistent elevated levels. The lowest value achieved in TP is 0.131 mg/l on 31 May. The lowest value of OP was 1 day later on 1 June with a value of 0.079 mg/l. The event measurements displayed significantly higher concentration peaks in all parameters compared to the daily mixed samples. Notably, in event 6, the concentration of TP exceeded the associated daily mixed sample by up to 0.46 mg/l, while the concentration of OP showed an increase of up to 0.32 mg/l. The largest difference of 0.16 mg/l in PP was observed during event 7. Moreover, the maximum sediment concentration during event 9 exceeded the value of the mixed sample by a factor of 30. This highlights the importance of considering specific events in monitoring and assessing phosphorus levels in water systems (Fig. 8). Furthermore, to assess how mean values from the event samples compare to the mean values of the daily mixed samples the weighted daily samples and the weighted event samples were compared. Since the daily mixed samples span exactly 24 h starting from 00:00 until 23:59, they were weighted against the exact measurement period of the event-based results. For example, event 1 started on May 21 at 16:40 and continued until May 22 at 16:40. The daily mixed sample from 21 May was given a weight of 30.5%, and the daily mixed sample of the 22 May was given a 69.5% weight, to represent both days in the comparison with the 24-h period of the event sample. Overall, the results suggest that at Gauge Soltfeld, the event measurements displayed notably higher concentration peaks compared to the daily mixed samples. These findings highlight the importance of considering specific events in monitoring and assessing phosphorus levels in water systems (Fig. 8).

A substantial portion of SS and PP transport in catchments occurs during infrequent storm events during small fraction of the entire year. Consequently, an intensive monitoring approach is crucial to accurately capture these transient periods of elevated transport, highlighting the need to monitor them closely (Kronvang et al. 1997; Kronvang and Bruhn 1996). Employing event-based sampling enhances the precision of TP annual load estimations in some catchments (Lessels and Bishop 2015). This however proves to be comparatively subdued in catchments characterized by minor relief and reduced annual rainfall (Lessels and Bishop 2015). In lowland catchments with limited slope, the applicability might be constrained, and focus instead on short, transient periods characterized by greater transport (Kronvang et al. 1997; Kronvang and Bruhn 1996).

**Fig. 11:**
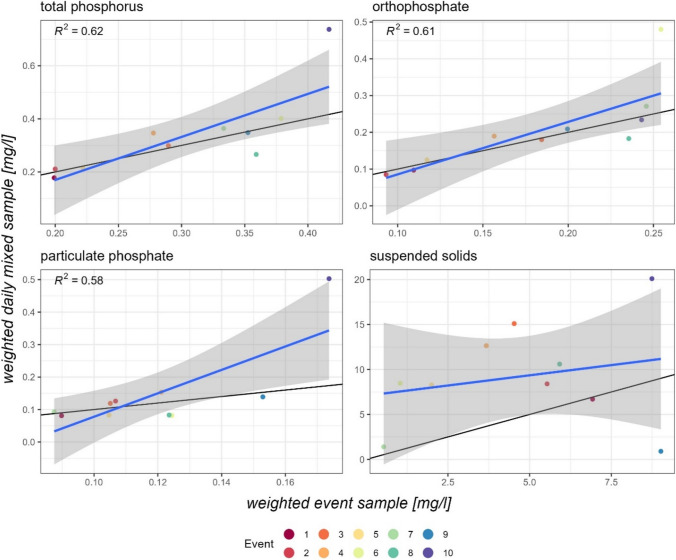
Comparison of the weighted daily mixed samples with the weighted event samples (regression line in blue, 1:1 line in black, grey shows the confidence interval)

Regarding the phosphorus fractions, the 24-h mean values of the event samples and the daily mixed samples demonstrated a significant agreement, as indicated by a significant relationship (*p* < 0.05). However, in terms of the SS samples, the mean values exhibited significant differences and did not show a significant correlation (Fig. 11 in the Appendix). The significant agreement observed in the phosphorus fractions between the event and daily mixed samples further strengthens the reliability of the data and suggests that while the daily mixed samples miss the significantly higher and lower short-term concentrations, the average phosphorus fraction is depicted accurately in most cases. However, the divergent SS mean values indicate the need for further investigation to understand the factors contributing to sediment variations and their implications for phosphorus dynamics in the study area. An additional monitoring to accurately depict the SS concentrations shortly after rain events in the form of event-based sampling would be beneficial.

## Conclusion

The analyses of phosphorus dynamics within the lowland catchment Kielstau showed an increase of concentrations during the last 15 and especially during the last 5 years. Seasonal variations in phosphorus concentrations, with peaks occurring during summer months followed by declines in winter, especially notable in TP and PP, were found. The long-term data indicated rising concentrations, notably in TP and PP that were linked to point sources like wastewater treatment plants and diffuse inputs from the rural landscape during heavy rainfall. The quantitative and temporal changes in the phosphorus concentrations in the event-based sampling showed very different responses to precipitation in the different events and sampling points. Heavy precipitation events were observed, leading to short-term concentration peaks above the daily averages. Differences in the reaction time of Gauges Soltfeld and Moorau were identified and may be attributed to catchment size, precipitation distribution, and soil moisture conditions. Catchment area emerged as a determining factor influencing reaction and response times following precipitation events. This highlights the necessity for monitoring approaches with higher resolutions in smaller catchments, with their faster response times compared to bigger catchments. Concentration patterns suggest a more complex, less predictable system in the smaller catchment compared to a dilution effect in the larger catchment. This indicates a greater need for event-based sampling in smaller catchments, where heavy precipitation events have stronger immediate impacts. It is notable that not just both sampling points, but also each individual event greatly differs from the others in terms of behavior and characteristics so that a general prediction about the course of future events is difficult.

With regard to the applied methodology, the event-based sampling could be refined to be dependent on flow velocity instead of water level changes, to capture the small peaks more effectively. Event-based sampling based on water level may otherwise lead to missing a portion of these peaks. A direct comparison of different event-based sampling methods would lead to more definite information. While the logarithmic sampling approach is useful to capture changes at the beginning of the event at a high resolution, a sampling approach using equidistant timing can be recommended for the analysis of auto- and cross-correlations as well as lag times. The precipitation event-based measurements of the phosphorus concentrations with daily mixed samples at the sampling point at Gauge Soltfeld suggest that while the event measurements displayed notably higher short-term concentration peaks, the average concentrations are comparable. This highlights the importance of considering specific events in lowland catchments in monitoring and assessing phosphorus levels in rivers. The data indicates that an additional event-based monitoring is particularly beneficial with regard to measuring SS concentrations. Especially with methodological refinements, event-based sampling enhances the efficacy of capturing short-term concentration peaks.

Given the availability of the unique, detailed and long-term daily data set of water quality for the Kielstau catchment, modeling can help to further analyze factors influencing phosphorus dynamics, migration pathways for phosphorus, and suspended solids. In other lowland catchments where such detailed daily measurements are not available, event-based sampling is more relevant for comprehensive data collection.

## Data Availability

The data is available from the corresponding author upon reasonable request.

## Appendix

Figure 9, 10 and 11
